# H3K4me2 marks the enhancer: Enhancer logic in the zebrafish embryo

**DOI:** 10.1371/journal.pbio.3003257

**Published:** 2025-07-11

**Authors:** Noura Maziak, Juan M. Vaquerizas

**Affiliations:** 1 MRC Laboratory of Medical Sciences, London, United Kingdom; 2 Institute of Clinical Sciences, Faculty of Medicine, Imperial College London, London, United Kingdom

## Abstract

At a key point in development, the embryo activates its genome: a shift that is largely coordinated by maternally derived factors. A new study in PLOS Biology identifies H3K4me2-marked enhancers in zebrafish that function independently and mirror the gamete state.

In the earliest stages of development, the embryo relies on maternal instructions—proteins and RNAs deposited into the egg during oogenesis—that direct essential processes such as cell division and pattern formation. This maternal control is transient. As development progresses, regulatory control shifts to the embryo in a process known as the maternal-to-zygotic transition, when the embryo begins activating its own genetic program. To achieve this shift, the embryo undergoes zygotic (embryonic) genome activation (ZGA): the onset of genome-wide transcription that is essential for continued development and differentiation, involving the coordinated expression of thousands of genes [1].

Across taxa, this hand-off is driven in large part by maternally supplied pioneer transcription factors. These proteins are unique in their ability to bind and open regulatory regions of the genome, enabling access for the transcriptional machinery and launching the embryo’s first gene expression programs. While proper ZGA is critical to the embryo, its underlying mechanisms are complex and variable. In *Drosophila*, the pioneer transcription factors Zelda, GAF, and CLAMP have been well characterized for their roles in orchestrating ZGA [2–4]. However, many genes are activated independently of these factors. Interestingly, the histone variant H2A.Z is enriched at Zelda-independent genes, and the loss of its chaperone is embryonically lethal highlighting multiple regulatory pathways exist [5]. In humans and mice, the embryonically expressed transcription factors DUX4 and Dux, respectively, have been identified as key regulators of ZGA [6]. Surprisingly, in mice, embryos lacking Dux alone still develop normally and are fertile, raising questions about its essentialness [7]. This apparent paradox in mice was resolved by the discovery of Obox4’s role, another transcription factor that functions alongside Dux [8]. In this system, only the combined loss of Dux and Obox4 results in impaired embryonic development. In zebrafish, the focus of the present study, ZGA is largely governed by the pioneer factors Nanog, Pou5f3 (an Oct4 homolog), and Sox19b (a Sox2 homolog), collectively termed NPS [9]. These factors can act partially redundantly to each other and initiate chromatin opening and activate gene expression, yet even triple-mutant embryos can still activate a subset of genes [10]. Together, these findings illustrate that ZGA is not a monolithic event but rather a multilayered process with many parallel pathways.

In a new study, Hurton and colleagues [11] extend the view by characterizing two distinct subclasses of enhancers in the early zebrafish embryo. By profiling 10 histone modifications at the end of ZGA, Hurton and colleagues were able to distinguish regions that control gene activity—specifically separating gene promoters from enhancers. Among the enhancers, the majority were marked by H3K4me1, but a bona fide, distinct subset was enriched for H3K4me2, allowing classification into two groups. A particularly compelling aspect of the study was the authors’ use of a reporter assay to test representative elements from both classes. This approach demonstrated that both H3K4me1- and H3K4me2-marked regions possess enhancer activity. Of note, a subset of reporters from both classes also exhibited some promoter-like activity. This observation raises interesting questions about how multiple regulatory potentials can be encoded within a single genomic region.

The authors then turned to characterizing how the chromatin landscape differs between the two enhancer classes. In contrast to classical H3K4me1-marked enhancers, which largely depend on the maternal pioneer factors NPS for activation, H3K4me2 enhancers did not lose accessibility in an NPS mutant background. Because H3K4me2-marked enhancers were initially identified based on their chromatin similarity to promoters, and because certain hypomethylated promoters are known to gain accessibility through incorporation of H2A.Z placeholder nucleosomes, the authors asked whether these enhancers might share similar epigenetic features. They found that H3K4me2 enhancers, in contrast with H3K4me1 enhancers, are both hypomethylated and enriched for H2A.Z. Additionally, the majority of H3K4me2 enhancer-regions have equivalent hypomethylation in both eggs and sperm suggesting that they are epigenetically “bookmarked”, carrying a memory of germline regulatory history. Collectively, these findings reveal the existence of parallel enhancer pathways in the early embryo, each driven by distinct regulatory factors, operating through different chromatin features, and shaped by separate histories of genomic activity (Fig 1).

**Fig 1:**
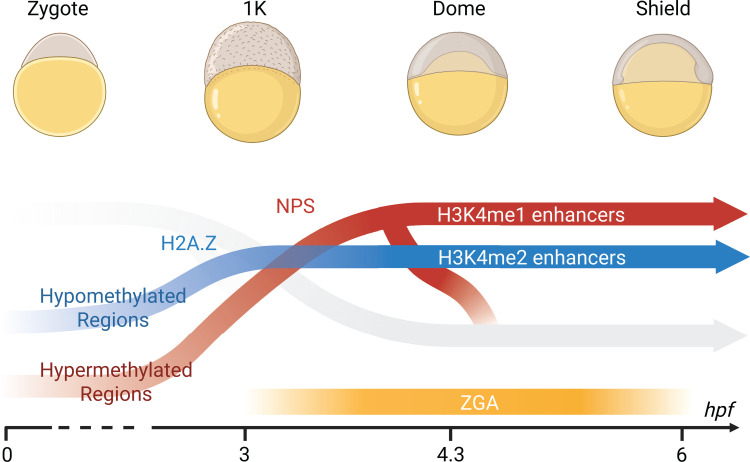
Parallel paths to enhancer activation during the maternal-to-zygotic transition in zebrafish. Two distinct classes of enhancers emerge as the embryo transitions from maternal to zygotic control. H3K4me1-marked enhancers (red) are established through NPS pioneer factor activity and arise in hypermethylated regions. In contrast, H3K4me2-marked enhancers (blue) originate from mostly hypomethylated regions and become active independently of NPS. These enhancers are also associated with H2A.Z incorporation. Together, they represent parallel regulatory trajectories converging on zygotic genome activation (ZGA). Developmental stages and time in hours post-fertilization (hpf) are indicated above and below.

This work opens several exciting avenues for future exploration in the field. For instance, which transcription factors might regulate these newly identified H3K4me2-marked enhancers? Analysis by the authors points to several intriguing candidates, including E2F factors and the well-known chromatin organizer CTCF. Another potentially interesting avenue is to examine whether these enhancers are conserved at the sequence level, particularly in comparison to classical NPS-dependent enhancers. Beyond these questions, much remains to be uncovered about the broader regulatory landscape in which these enhancers operate—including which genes they control and how enhancer activity is modulated over time. These investigations could refine models of enhancer function and deepen our understanding of the more mechanistic aspects of genome regulation.

Finally, this study illustrates that, while the mechanisms of ZGA vary across species, certain themes recur. For instance, H2A.Z, previously implicated in Zelda-independent regulation in Drosophila [5], now appears again in zebrafish at NPS-independent enhancers. These parallels underscore that although ZGA is resolved differently across each organism, many of the underlying challenges the embryo must overcome are shared. Such findings deepen our appreciation for the flexibility and constraints of genome activation and set the stage for uncovering how different species innovate on a common developmental challenge.

## Abbreviations:

NPS: Nanog, Pou5f3, Sox19b,
ZGA: zygotic genome activation
